# Efficient Inverted Index Compression Algorithm Characterized by Faster Decompression Compared with the Golomb-Rice Algorithm

**DOI:** 10.3390/e23030296

**Published:** 2021-02-28

**Authors:** Andrzej Chmielowiec, Paweł Litwin

**Affiliations:** 1 The Faculty of Mechanics and Technology, Rzeszow University of Technology, Kwiatkowskiego 4, 37-450 Stalowa Wola, Poland; 2 The Faculty of Mechanical Engineering and Aeronautics, Rzeszow University of Technology, Powstańców Warszawy 8, 35-959 Rzeszow, Poland; plitwin@prz.edu.pl

**Keywords:** inverted index compression, Golomb-Rice coding, runs coding, sparse binary sequence compression

## Abstract

This article deals with compression of binary sequences with a given number of ones, which can also be considered as a list of indexes of a given length. The first part of the article shows that the entropy *H* of random *n*-element binary sequences with exactly *k* elements equal one satisfies the inequalities $k\log_{2}\left(0.48\cdot n/k\right)<H<k\log_{2}\left(2.72\cdot n/k\right)$. Based on this result, we propose a simple coding using fixed length words. Its main application is the compression of random binary sequences with a large disproportion between the number of zeros and the number of ones. Importantly, the proposed solution allows for a much faster decompression compared with the Golomb-Rice coding with a relatively small decrease in the efficiency of compression. The proposed algorithm can be particularly useful for database applications for which the speed of decompression is much more important than the degree of index list compression.

## 1. Introduction

Our research is aimed at solving the problem of efficient storage and processing of data from production processes. Since the introduction of control charts by Shewart [1,2], quality control systems have undergone a continuous improvement. As a result of the ubiquitous computerization of production processes, enterprises today collect enormous amounts of data from various types of sensors. With annual production reaching tens or even hundreds of millions of items, this implies the need to store and process huge amounts of data. Modern factories with such high production capacity are highly automated, thus being equipped with a substantial number of sensors. Today, the number of sensors installed in a production plant usually ranges between $10^{3}$ (a medium sized factory), and $10^{5}$ (a very large factory). We can, thus, describe the state of a factory at time *t* as a sequence of bits $S_{t}=\left(s_{1,t},s_{2,t},\ldots ,s_{m,t}\right)$, where the bit $s_{i,t}$ indicates if the production process in a given place proceeds correctly. By noting down states $S_{t_{j}}$ at times $t_{1}<t_{2}<\cdots <t_{r}$, we obtain a matrix of binary states:

$$\begin{array}{c} \left(s_{i,t_{j}}\right)=\left(\begin{array}{cccc} s_{1,t_{1}} & s_{2,t_{1}} & \cdots & s_{m,t_{1}}\\ s_{1,t_{2}} & s_{2,t_{2}} & \cdots & s_{m,t_{2}}\\ \vdots & \vdots & \ddots & \vdots \\ s_{1,t_{r}} & s_{2,t_{r}} & \cdots & s_{m,t_{r}} \end{array}\right), \end{array}$$

which illustrates the state of production processes in a given time period. The data from the matrix of measurements can be analyzed, e.g., in order to detect the relationship between the settings of production processes and the number of defective products [3]. It should be noted, however, that, by saving sensor readings every 0.1 s, a factory collects from $2.5\cdot 10^{10}$ to $2.5\cdot 10^{12}$ bits of new data monthly (3÷300 (GB)). Such large monthly data increases require the application of appropriate compression methods enabling quick access to information stored in a database. Note that this type of data is characterized by a significant disproportion between the number of zeros and ones. For example, if 0 means no process defects/no failure, and 1 denotes a defect/a failure, then a typical production process database usually stores binary sequences with a large number of zeros and a small number of ones. In practice, a need arises to store a list of indexes containing the numbers of detectors and times for which specific deviations have been observed. Such databases naturally contain lists (or matrices) of indexes comprising information about various types of abnormalities. This is why inverted index methods, which today are widely used in all kinds of text document search engines [4,5,6], provide a workable solution to the problem of efficient storage of such databases. In its simplest form, the database comprises only zeros (OK) and ones (FAIL), which means that there is basically only one word to index (FAIL). However, we can easily imagine that the database also stores error types, in which case it is necessary to index many words—one word for every error code. Such a database can effectively store information about mechanical failures, overheating, thermal damage, product defects, downtime, etc. This is why the inverted index method proves useful for storing data on various failures and irregularities. In addition, it must be stressed that the data stored in the database will not be modified. This means that the focus must be on maximizing the degree of compression, while ensuring high speed data search (decompression). These two postulates underlie the construction of the algorithm presented in this article.

Currently there are three main approaches to inverted indexing compression.

1. Methods for compressing single indicators, including: Shannon-Fano coding [7,8], Huffman coding [9], Golomb-Rice coding [10,11], Gamma and Delta coding [12], Fibbonaci coding [13], (S,C)-dense coding [14], and Zeta coding [15].
2. Methods for compressing entire lists, including Elias-Fano method [16,17], interpolation methods [18], Simple-9, Relative-10, and Carryover-12 [19].
3. Methods for compressing many lists simultaneously, which mainly adapt and increase the potential of the methods listed above.

The article by Pibiri and Venturini [20] gives an extensive review of inverted index compression algorithms. Some publications deal with efficient decompression and compare the speed of individual algorithms in various environments [19,21,22]. As mentioned earlier, our research looks into the problem of compressing databases storing binary information about the status of manufactured items or even the entire production plant. The vast majority of this kind of data can be represented by binary sequences containing a small number of ones and a large number of zeros. When solving such problems, it is often assumed that the resulting sequences are random sequences. The problem of coding sparse binary sequences is often raised in the literature, for example in the book by Solomon [23]. One of the first algorithms was proposed by Golomb [10], who introduced quotient and remainder coding for the distance between successive occurrences of ones. Then, Gallager and van Voorhis [24] derived a relationship that allows for optimal selection of compression parameters for Golomb codes. Somasundaram and Domnic [25] proposed an extended Golomb code for integer representation. The method proposed by References [26,27] and developed, among others, by Fenwick [28] is a special case of Golomb coding. Since these two types of coding are interrelated, they are often referred to as Golomb-Rice codes. The work on the appropriate selection of parameters for these codes was later continued, among others, by Robinson [29] and Kiely [30]. On the other hand, Fraenkel and Klein [31] proposed completely different methods for coding sparse binary sequences by combining Huffman coding with the new numeral systems. Another algorithm that can be considered as useful for coding these types of sequences is the prefix coding proposed by Salomon [32]. In turn, Tanaka and Leon-Garcia [33] developed efficient coding of the distance between ones when the probability of their occurrence varies. It is also worth mentioning the work by Ferragina and Venturini [34], who proposed a new method of creating word indexes.

The works of Trotman [21] and Zang [35] were the motivation for choosing Golomb-Rice as the reference algorithm. Trotman [21] compares set sizes and decompression times using the Golomb-Rice, Elias gamma, Elias delta, Variable Byte, and Binary Interpolative Coding algorithms. These compression techniques were studied in the context of accessing the Wall Street Journal collection. The Golomb-Rice algorithm is reported as a second in the compression ratio category (Binary Interpolative Coding is slightly better), and the lowest compression ratio is achieved by Variable Byte. The shortest decompression time was obtained using Variable Byte Coding. The Golomb-Rice algorithm ranks second in the decompression time category. It is worth it to note that Binary Interpolative Coding in this category is clearly weaker than the other methods. In the work of Zang [35], the size of sets and the decompression speed of the Golomb-Rice, Variable Byte, Simple9, Simple16, and PForDelta algorithms were analyzed. The task concerned decompression of inverted index files in search engines. In the analysis, the Golomb-Rice algorithm obtains the smallest sizes of the compressed sets, but, in the case of decompression time, it is weaker than the competition (only Variable Byte achieves longer decompression times).

When we consider the problem of storing and processing large data sets, e.g., data collected in enterprises operating according to the Industry 4.0 framework, both the size of the saved files and the access time are important. The Golomb-Rice algorithm is characterized by a high compression ratio and a high decompression speed, lower only than the algorithms that use vector instructions available in modern processors. However, such instructions are not implemented in all devices of the industrial Internet of Things solutions.

In the next parts of the article we present: a theorem on the entropy of random binary sequences with a given number of zeros and ones, a new algorithm for coding sparse binary sequences, and an analysis of the results of the implemented algorithm. We decided to compare the algorithm proposed in the article with an algorithm using Golomb-Rice coding and the DEFLATE algorithm implemented in the ZLIB library, which was developed based on the foundations laid by Lempel and Ziv [36,37]. At this point, it should be stressed that the main point of reference of the presented results is the Golomb-Rice algorithm, which proved to be most efficient as far as compression is concerned but leaves much to be desired in terms of speed.

## 2. Materials and Methods

At the beginning of this section, we analyze the entropy of *n*-element binary sequences consisting of exactly *k* ones and n−k zeros. Let *X* be a random variable defined in space:

$$\begin{array}{c} \Omega =\left\{x:\;x=\left(x_{1},\ldots ,x_{n}\right),\;x_{1}+\cdots +x_{n}=k,\;x_{i}\in \left\{0,1\right\}\right\}, \end{array}$$

for which all events are equally probable. Consequently, for every x∈Ω, the following holds:

$$\begin{array}{c} P\left(X=x\right)=P_{n,k}=\frac{k!(n-k)!}{n!}=\frac{k!}{(n-k+1)\cdots (n-1)n}, \end{array}$$

which, directly from the definition of entropy, leads to:

$$\begin{array}{c} H\left(X\right)=-\log_{2}\left(P_{n,k}\right). \end{array}$$

Later in the article, we will show that the asymptotic entropy of a random variable *X* can be expressed by $H\left(X\right)=\Theta \left(k\log_{2}\left(\frac{n}{k}\right)\right)$. This is of great practical importance because it means that such sequences cannot be encoded more efficiently than with $c\cdot k\log_{2}\left(\frac{n}{k}\right)$ bits, where *c* is a constant. Looking at the entropy H(X) from the perspective of the encoding of natural numbers, we can conclude that it is on the same level as the entropy of *k* numbers not greater than n/k. This observation refers to a real situation where *k* ones are randomly distributed in an *n*-element sequence, while the average distances between them are n/k. This very observation forms the basis for the compression algorithm presented later in the article. The algorithm is based on the encoding of the distance between adjacent ones on words of a fixed width equal approximately to $\log_{2}\left(\frac{n}{k}\right)$. At this point, we must strongly emphasize that the case analyzed in the article is fundamentally different from a situation where a random variable *Y* equals 1 with probability p=k/n. Entropy is given by the well-known expression $H\left(Y\right)=-p\log_{2}\left(p\right)-\left(1-p\right)\log_{2}\left(1-p\right)$, while the *n*-element sequences generated using this random variable do not have a fixed number of ones equal to *k*. A very accurate estimate of the value of H(Y) was proposed by Mascioni [38]. His inequalities could also be used to estimate the entropy H(X), but, unfortunately, they lead to much less transparent and non-intuitive relationships. This is why, in this section, we present our own original estimate of the value of H(X).

The value $H\left(X\right)=-\log_{2}\left(P_{n,k}\right)$ is the number of bits of information carried by the survey. Unfortunately, this formula is not a intuitional reflection of the actual size of the coded information. That is why it is our goal to express H(X) using more intuitive formulas.

An element that we will use is the Stirling’s formula given by Robbins [39]:

**Theorem** **1** (Stirling’s formula)**.** *If n is a natural number, then:*

(1) $$\begin{array}{c} n!=\sqrt{2\pi n}{\left(\frac{n}{e}\right)}^{n}e^{\lambda_{n}}, \end{array}$$

*where $\frac{1}{12n+1}<\lambda_{n}<\frac{1}{12n}$.*

Having the above in mind, we are ready to prove the main theorem about the entropy of *n*-element binary sequences with exactly *k* elements other than zero.

**Theorem** **2.** *If X is a random variable with domain:*

$$\begin{array}{c} \Omega =\left\{x:\;x=\left(x_{1},\ldots ,x_{n}\right),\;x_{1}+\cdots +x_{n}=k,\;x_{i}\in \left\{0,1\right\}\right\}, \end{array}$$

*$1\leq k<\frac{n}{2}$ and probability P(X=x) is the same for every sequence, and then entropy H(X) satisfies the following inequalities:*

(2) $$\begin{array}{c} k\log_{2}\left(\frac{0.48\cdot n}{k}\right)<H\left(X\right)<k\log_{2}\left(\frac{2.72\cdot n}{k}\right). \end{array}$$

**Proof.**  The proof consists of two parts. First, it is shown that the entropy H(X) is bounded from above:

$$\begin{array}{cc} H(X) & =\log_{2}\frac{(n-k+1)\cdots (n-1)n}{k!}\\ \; & <\log_{2}\frac{n^{k}}{k!}\\ \; & <\log_{2}\left(n^{k}{\left(\frac{e}{k}\right)}^{k}\cdot e^{-\frac{1}{12k+1}}\cdot {\left(2\pi k\right)}^{-\frac{1}{2}}\right)\\ \; & =k\log_{2}\left(\frac{en}{k}\cdot e^{-\frac{1}{12k^{2}+k}}\cdot {\left(2\pi k\right)}^{-\frac{1}{2k}}\right). \end{array}$$

The property of function $e^{x}$ shows that $e^{-\frac{1}{12k^{2}+k}}<1$, while, based on elementary facts about the limits of numerical sequences, we know that ${\left(2\pi k\right)}^{-\frac{1}{2k}}<1$. Consequently, it follows that:

$$H\left(X\right)<k\log_{2}\left(\frac{en}{k}\right)<k\log_{2}\left(\frac{2.72\cdot n}{k}\right).$$

In the second part of the proof, we show that the entropy H(X) is bounded from below:

$$\begin{array}{cc} H(X) & =\log_{2}\frac{(n-k+1)\cdots (n-1)n}{k!}\\ \; & >\log_{2}\frac{{\left(n-k+1\right)}^{k}}{k!}\\ \; & >\log_{2}\left({\left(n-k+1\right)}^{k}{\left(\frac{e}{k}\right)}^{k}\cdot e^{-\frac{1}{12k}}\cdot {\left(2\pi k\right)}^{-\frac{1}{2}}\right)\\ \; & =k\log_{2}\left(\frac{e(n-k+1)}{k}\cdot e^{-\frac{1}{12k^{2}}}\cdot {\left(2\pi k\right)}^{-\frac{1}{2k}}\right). \end{array}$$

For $k<\frac{n}{2}$ and k≥1, we have $\frac{e(n-k+1)}{k}>\frac{en}{2k}$. The property of function $e^{x}$ shows that $e^{-\frac{1}{12k^{2}}}>e^{-\frac{1}{12}}>0.92$, while, based on elementary facts about the limits of numerical sequences, it follows that ${\left(2\pi k\right)}^{-\frac{1}{2k}}>0.39$. By combining all the above inequalities, we finally obtain:

$$\begin{array}{c} H\left(X\right)>k\log_{2}\left(\frac{0.48\cdot n}{k}\right). \end{array}$$

This completes the proof. □

**Figure 1:**
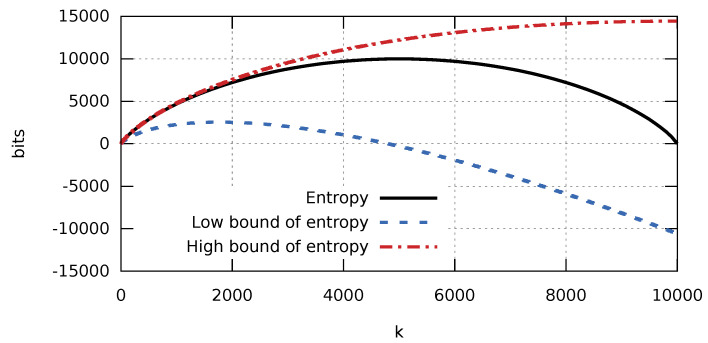
Graph of entropy and its upper and lower bounds in the function of the number of ones for a sequence consisting of $10^{4}$ elements for $k\in [0,10^{4}]$.

**Figure 2:**
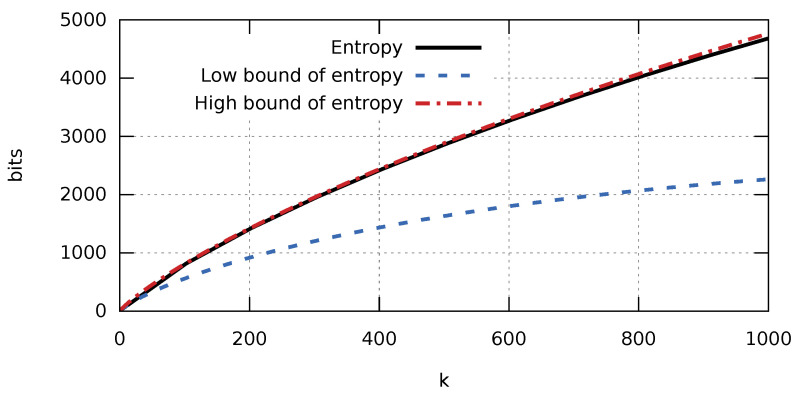
Graph of entropy and its upper and lower bounds in the function of the number of ones for a sequence consisting of $10^{4}$ elements for $k\in [0,10^{3}]$.

It is worth noting how accurate the obtained estimates are. Figure 1 shows a complete graph of the entropy H(X) and its bounds arising from Theorem 2. A graphical interpretation of these relationships clearly shows that the lower bound strongly deviates from the value of entropy, whereas the graph of the upper bound almost converges with the graph of entropy for a fairly large range of *k* values. Figure 2 shows graphs for *k* bound by $\frac{1}{10}n$. Therefore, we can expect that a compression algorithm developed based on estimate (2) will have greater compression efficiency for small *k* values. This matches our expectations, as the degree in which a database of a production process is filled with various errors and failures should be relatively low (low k/n value).

The estimate obtained in Theorem 2 also allows for a good approximation of the value of Newton’s binomial coefficients. Our observations show that the entropy can be well expressed by formula $H\left(X\right)\approx k\log_{2}\left(\frac{2.72\cdot n}{k}\right)$. Based on the fact that $H\left(X\right)=-\log_{2}\left(P_{n,k}\right)$, we obtain the following approximation of binomial coefficients for small *k* values:

(3) nk=Pn,k=e−H(X)≈eklog22.72·nk.

Although the approximation is not as good as that proposed by Mascioni [38], the formula is so compact and elegant that is seems worthy of attention.

Underlying the new algorithm is the Golomb-Rice coding [10,26,27], which is still used in many applications that require efficient data compression. It is vital for modern image coding methods [40,41], video compression [42,43], transmission coding in wireless sensor networks [44], compression of data from exponentially distributed sources [45], Gaussian distributed sources [46], and high variability sources [47]. Moreover, it is used for compressing data structures, such as R-trees [48] and sparse matrices [49]. The universality and versatility of Golomb-Rice codes results from the fact that with appropriate selection of parameters the level of compression is very close to the level of entropy. Figure 5 shows how close the entropy curve and the Golomb-Rice coding compression curve are to each other. In fact, the only defect of Golomb-Rice coding is the variable length of the codeword, which, in the case of software applications, negatively affects the time of compression and decompression. Bearing in mind the applications mentioned in the introduction, we decided to propose a new algorithm with a compression level, similar to that offered by Golomb-Rice coding, but with faster decompression.

Let us consider a binary sequence compressed with the use of Golomb-Rice coding with module $m=2^{2}=4$:

(4) $$\begin{array}{c} 0\;0\;0\;0\;0\;1\;1\;0\;0\;0\;0\;0\;1\;0\;0\;1\;0\;0\;0\;0. \end{array}$$

For this particular sequence, it is necessary to encode a sequence of integers (5,0,5,2,4) and transform it under the Golomb-Rice method into the following sequence of bits:

(5) $$\begin{array}{c} \underset{5}{\underbrace{1\;|\;0\;|\;01}}\;|\;\underset{0}{\underbrace{0\;|\;00}}\;|\;\underset{5}{\underbrace{1\;|\;0\;|\;01}}\;|\;\underset{2}{\underbrace{0\;|\;10}}\;|\;\underset{4}{\underbrace{1\;|\;0\;|\;00}}. \end{array}$$

Vertical bars | in the code above mark places in which it will be necessary to execute a conditional instruction. We will call them codeword divisions, while their number will be called the *number of codewords*. The general Golomb-Rice compression process is presented as Algorithm 1. Whenever used in the algorithm, ${\left(m\right)}_{2,n}$ denotes that the number *m* is coded in the binary system using exactly *n* bits. It should be noted that the optimal length *w* of the suffix is strictly dependent on the fraction k/n. The best estimate of this value was provided by Gallager and Voorhis [24]. In steps 4–5, the algorithm determines the length of the string of zeros to be encoded. Then, in steps 6–7, parameters for the codewords are determined. In step 8, the algorithm creates $q_{i}+1$ single-bit codewords and one *w*-bit codeword for the suffix.
**Algorithm 1:** Golomb-Rice compression of a binary sequence.
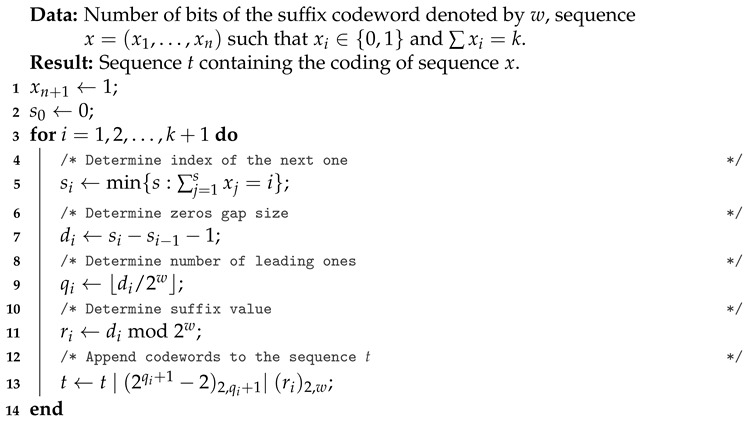


For the sake of completeness, Algorithm 2, in which the task is to decompress the Golomb-Rice coding, is also presented. Both Algorithm 1 and Algorithm 2 were used in the implementation designed for the comparative analysis of both methods.
**Algorithm 2:** Golomb-Rice decompression (zero series length encoding).
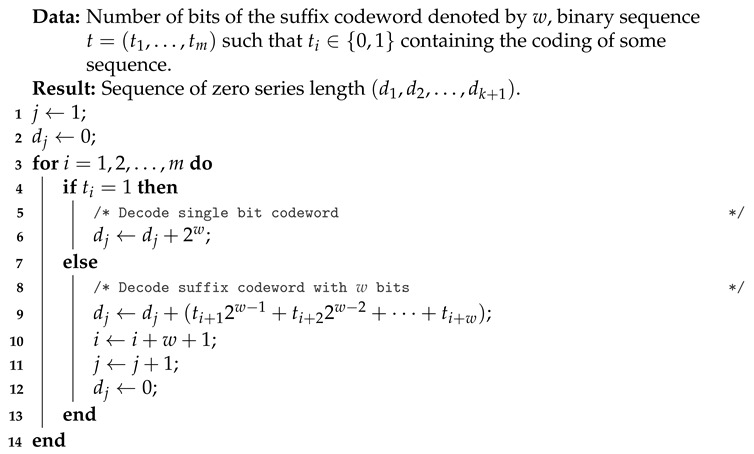


A large number of codewords negatively impacts on software performance. Unfortunately, the use of variable codeword length and unary coding implies that many more conditional instructions will have to be executed in Golomb-Rice coding compared to fixed codeword length codes. For example, if we use 3-bit fixed length words to encode sequence (4), we will obtain the following sequence:

$$\begin{array}{c} \underset{5}{\underbrace{101}}\;|\;\underset{0}{\underbrace{000}}\;|\;\underset{5}{\underbrace{101}}\;|\;\underset{2}{\underbrace{010}}\;|\;\underset{4}{\underbrace{100}}. \end{array}$$

There are far fewer places in the sequence in which it is necessary to execute a conditional instruction. Now, we will use the theorem proved in the previous section to develop a new binary sequence compression algorithm.

Theorem 2 clearly shows that the entropy of an *n*-element binary sequence containing *k* ones equals $\Theta \left(k\log_{2}\left(\frac{n}{k}\right)\right)$. This formula gives some intuition about potential coding with its binary representation being of similar size to the entropy. Expression $k\log_{2}\left(\frac{n}{k}\right)$ suggests that *k* numbers, each of them of order $\frac{n}{k}$, should be enough to code the sequence. But *k* is the number of ones in the sequence, and $\frac{n}{k}$ is the average distance between them. Therefore, coding the distances between ones seems to be the most natural way of coding this type of sequences. This observation forms the basis for a new compression algorithm presented further in this section. The proving of Theorem 2 was crucial to making this observation. In the next section, we will analyze the properties of the proposed algorithm and set out the conditions under which the proposed method performs better than the standard algorithms of lossless compression.

We define the algorithm for compression sparse binary sequences (AC-SBS) as a parameterized algorithm with the parameter being the bit size of the codeword. The analysis of the algorithm will show that, for a given number of ones in a sequence, there exists exactly one codeword length minimizing the size of the compressed sequence. However, this property will not be analyzed in this section and we will present a version of the algorithm allowing for the use of any codeword length for every bit sequence. We will use the following example to illustrate the compression algorithm. Let us assume that we want to compress sequence (4) using a 2-bit codeword. To start with, let us note that a 2-bit word allows us to code four symbols. Since we need a way to code any distance between adjacent ones, one symbol will have a special meaning. Therefore, we propose the following method of representing distance:

- distance of 0 is coded as $c\left(0\right)={\left(0\right)}_{2}=00$,
- distance of 1 is coded as $c\left(1\right)={\left(1\right)}_{2}=01$,
- distance of 2 is coded as $c\left(2\right)={\left(2\right)}_{2}=10$,
- distance of m>2 is coded as $l=\lfloor \frac{m}{3}\rfloor$ symbols ${\left(3\right)}_{2}=11$ ended with a symbol coding the number m−3l.

Moreover, in order to precisely mark the end of a sequence, we will always add one at the end, which will then be removed once the decoding is complete. Consequently, the following sequence:

$$\begin{array}{c} 0\;0\;0\;0\;0\;1\;1\;0\;0\;0\;0\;0\;1\;0\;0\;1\;0\;0\;0\;0\;\;\bar{1} \end{array}$$

is coded as:

$$\begin{array}{c} \underset{5}{\underbrace{11\;|\;10}}\;|\;\underset{0}{\underbrace{00}}\;|\;\underset{5}{\underbrace{11\;|\;10}}\;|\;\underset{2}{\underbrace{10}}\;|\;\underset{4}{\underbrace{11\;|\;01}}. \end{array}$$

This simple example illustrates the mechanism of the algorithm developed based on the analysis of entropy of random binary sequences. Now, we are ready to provide a formal description of the compression algorithm in a general case.

Let us compress an *n*-element binary sequence consisting of exactly *k* ones. In addition, we will assume that *w*-bit words are used for coding. Whenever used in the algorithm, ${\left(m\right)}_{2,w}$ denotes that the number *m* is coded in the binary system using exactly *w* bits (similarly to the example presented above, we allow the occurrence of leading zeros). Under these assumptions, the compression algorithm is presented in Algorithm 3.

**Algorithm 3:** AC-SBS compression of a binary sequence.

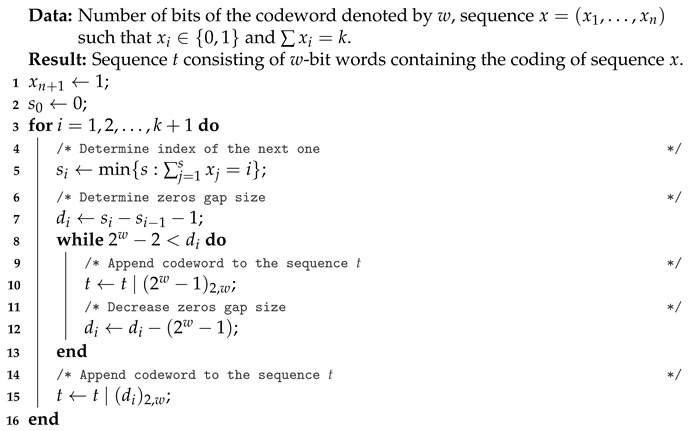



At the first step of the algorithm, we add one at the end of the sequence to be compressed. Next, in step 4, we set the number $s_{i}$, being in fact an index of the *i*-th one. In step 5, we set the number of zeroes between adjacent ones. The number $d_{i}$ denotes the number of zeros between ones with index i−1 and *i*. Then, in a 6−9 loop, we perform the main part of the algorithm, consisting of coding the number of zeros $\left\{d_{i}\right\}$.

Algorithm 4, on the other hand, is an AC-SBS decompression algorithm. This version of the algorithm was used in the comparative implementation. Like Algorithm 2, this one also returns the recovered lengths of a series of zeros. This is due to the fact that such a representation is much more useful for further work on the recovered data.
**Algorithm 4:** AC-SBS decompression (zero series length encoding).
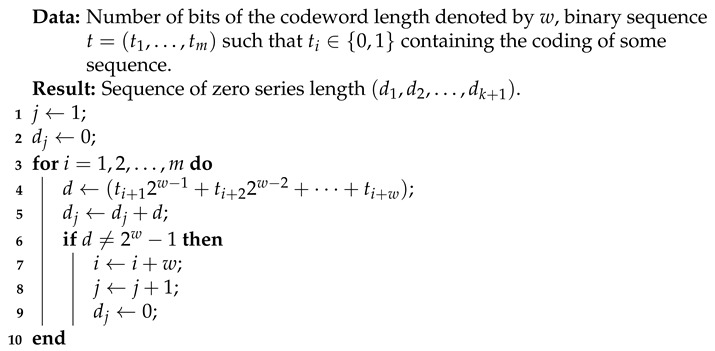


**Figure 3:**
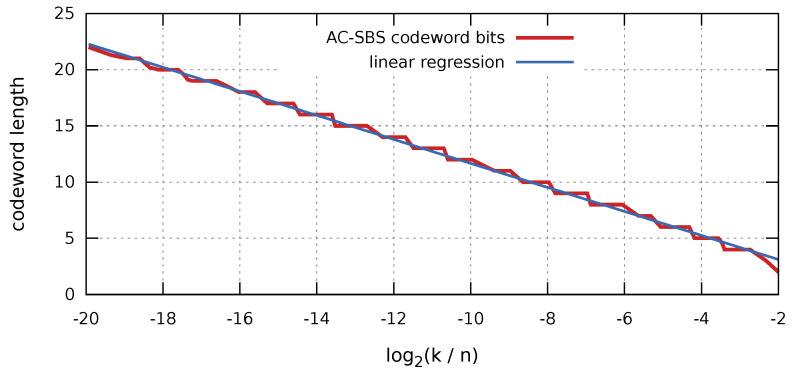
Graph of dependence of the optimal codeword length in the algorithm for compression sparse binary sequences (AC-SBS) algorithm on the value of $\log_{2}\left(k/n\right)$.

Algorithm 3 requires the length of the codeword to be used. Figure 3 shows the results of the statistical dependence of the optimal codeword length on the $\log_{2}\left(k/n\right)$ value. The regression line determined on the basis of the simulation has the form $l\left(k/n\right)=A\cdot \log_{2}\left(k/n\right)+B$, where A=−1.07,B=0.98, and leads to the following formula:

(6) $$\begin{array}{cc} w(k/n) & =\left\lfloor -1.07\cdot \log_{2}\left(\frac{k}{n}\right)+0.98+\frac{1}{2}\right\rfloor . \end{array}$$

The presented formula is practical, but perhaps it can be simplified and derived based on theoretical assumptions. As can be seen, the determination of w(k/n) values requires the knowledge of *k* and *n*, and, more precisely, their k/n ratio. For a given string of bits, these values can be simply computed by looking at the string. We can also predict the k/n value based on a sample of the analyzed data.

As mentioned earlier, the number of codewords to be read is a measure of the complexity of a decompression algorithm. In this case, the complexity $T_{GR}$ of the Golomb-Rice and $T_{AC}$ of the AC-SBS decompression algorithm can be approximated by the following formulas:

(7) $$\begin{array}{cc} T_{GR}\left(k,n\right) & =k\sum_{l=0}^{n-k}{\left(1-\frac{k}{n}\right)}^{l}\left(\frac{k}{n}\right)\left(w_{GR}+1+\left\lfloor \frac{l}{2^{w_{GR}}}\right\rfloor \right), \end{array}$$

(8) $$\begin{array}{cc} T_{AC}\left(k,n\right) & =k\sum_{l=0}^{n-k}{\left(1-\frac{k}{n}\right)}^{l}\left(\frac{k}{n}\right)\left(w_{AC}+\left\lceil \frac{l}{2^{w_{AC}}-1}\right\rceil \right), \end{array}$$

where $w_{GR}=w_{GR}\left(k/n\right)$ is optimal suffix length in Golomb-Rice algorithm, and $w_{AC}=w_{AC}\left(k/n\right)$ is optimal codeword length in AC-SBS algorithm. Due to the complexity of both formulas, it was decided that the exact number of codewords needed to encode a given binary sequence would be determined by the numerical experiment. The results of the research are presented below.

**Figure 4:**
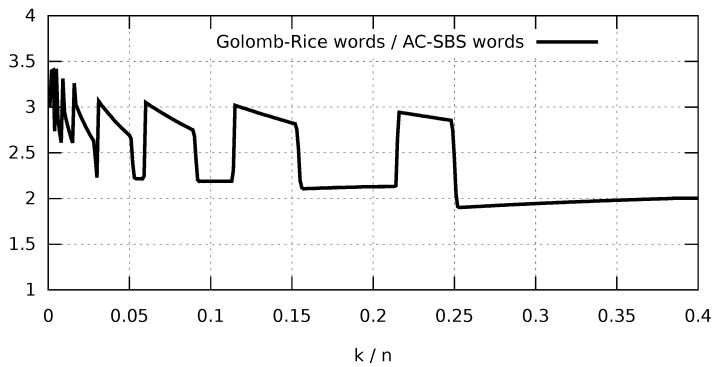
Average ratio of the number of Golomb-Rice codewords to the number of AC-SBS codewords.

In order to make an initial comparison of Golomb-Rice coding with AC-SBS coding, we analyzed the ratio of the number of codewords of both algorithms for various values of the *k* parameter. Figure 4 presents a graph of the ratio of the number of Golomb-Rice codewords to the number of AC-SBS codewords. The analysis was made for every $k\in \{100,200,300,\ldots ,4\cdot 10^{4}\}$ for 100 $10^{5}$-bit random sequences with the highest possible compression for every analyzed sequence (the optimal value was adopted for $w_{GR}$ and $w_{AC}$). The result clearly shows that the number of codewords in the case of AC-SBS is from three to two times lower compared with Golomb-Rice. The characteristic *jumps* in the graph are closely related to the change in the codeword length caused by the changing k/n ratio (the higher the ratio, the shorter the codewords). When the length $w_{AC}$ of the codeword in the AC-SBS algorithm increases, then we see a sharp increase in the graph. The sharp drops are caused by the change in suffix length $w_{GR}$ in the Golomb-Rice coding. In the next section, we will show correlation between the number of codewords and the rate of decompression of both algorithms.

## 3. Results

A comparative analysis of the AC-SBS algorithm was performed using two different hardware architectures. The first one was x86-64 with the AMD FX-6100 processor clocked at 1.573 GHz used for testing. The second was the ARM architecture with the Broadcom BCM2837 Cortex-A53 processor clocked at 1.200 GHz used for testing. In testing, we used our original implementation of the AC-SBS algorithm and the Golomb-Rice algorithm. The implementation has been made available in the GitHub repository at https://github.com/achmie/acsbs. All programs were compiled with the gcc compiler (version 9.3.0) with the -O2 optimization option turned on. In addition, we compared the obtained results with one of the fastest and most commonly used general compression libraries—ZLIB. The algorithms were selected to show the profound difference in compression efficiency and performance rate in the case when the compressed binary sequences have a large disproportion between the number of ones and zeros. Another advantage of this approach is that it shows that the efficiency of our original implementation does not differ a lot from the commonly used software.

The proposed compression algorithm was tested by means of compressing sparse binary sequences. The sequence consisted of $10^{6}$ elements. We generated random sequences with number of ones (*k*) ranging from 1 to $2.5\cdot 10^{5}$. For every *k*, $10^{3}$ random binary sequences were generated which were then compressed. The length of the compression word ranged from 2 to 12 bits. The purpose of our analysis was to estimate the size of the compressed sequence, as well as its decompression time. The results were then compared with the sequence size and decompression time obtained using the DEFLATE algorithm implemented in the ZLIB library (further referred to as the zlib-deflate algorithm) and Golomb-Rice coding. Moreover, the size of the compressed sequences was also compared with the theoretical level of entropy for a given type of sequence. Below, we will discuss the results and present graphs of sequence sizes and decompression times.

**Figure 5:**
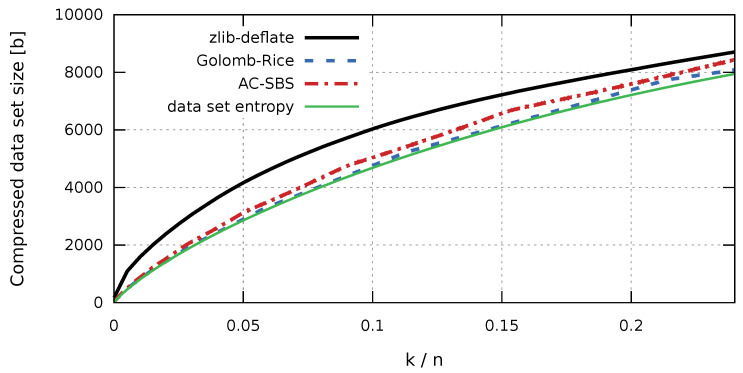
Sizes of compressed test sequences.

The first stage of our analysis is to compare the size of sequences compressed using the AC-SBS algorithm with sequences compressed using zlib-deflate, Golomb-Rice coding, and sequence entropy. The results shown in Figure 5 present an estimate of the sequence size for k/n from 0–0.25.

The graph presented in Figure 5 clearly indicates that the largest size are sequences compressed using the popular zlib-deflate algorithm. On the other hand, the size of the sequences obtained by AC-SBS compression is slightly larger than the sequences encoded by the Golomb-Rice method, which in turn gives a size slightly larger than the entropy of the sequences. Table 1 shows a comparison of the relative size of sequences obtained by the mentioned compression methods in relation to the entropy for selected k/n values.

It is worth noting that, for k/n not exceeding 0.02, the difference between the AC-SBS and Golomb-Rice methods does not exceed 7%, while the sequence compressed with the zlib-deflate method can be up to five times larger. The k/n values in this range are particularly important for storing information about the number of defective products in mass production, e.g., in the glass industry [3].

**Figure 6:**
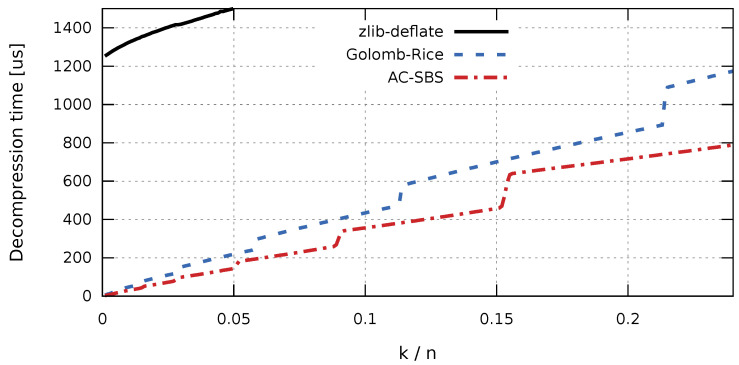
Decompression time of test sequences (ARM architecture).

**Figure 7:**
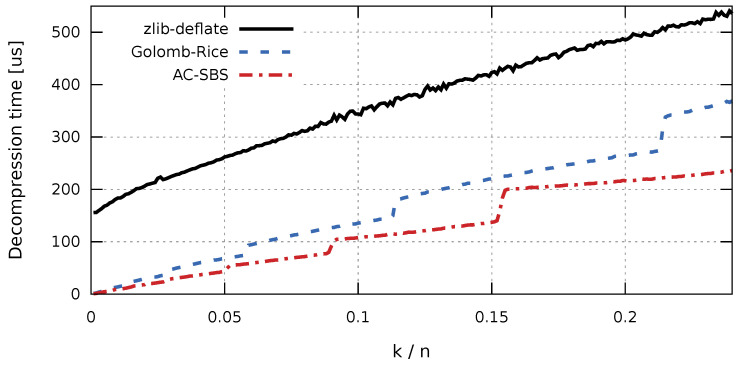
Decompression time of test sequences (x86 architecture).

**Figure 8:**
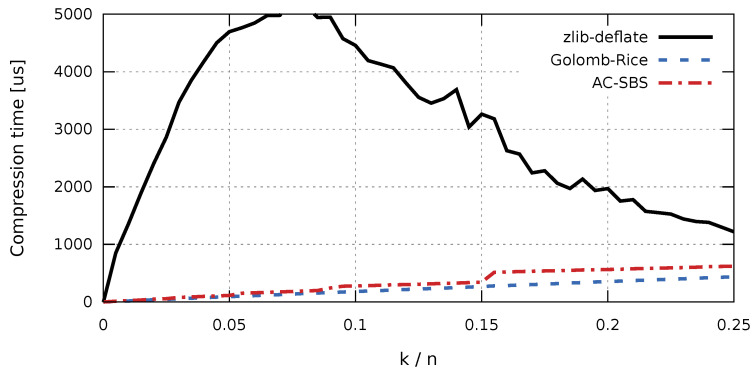
Compression time of test sequences (ARM architecture).

**Figure 9:**
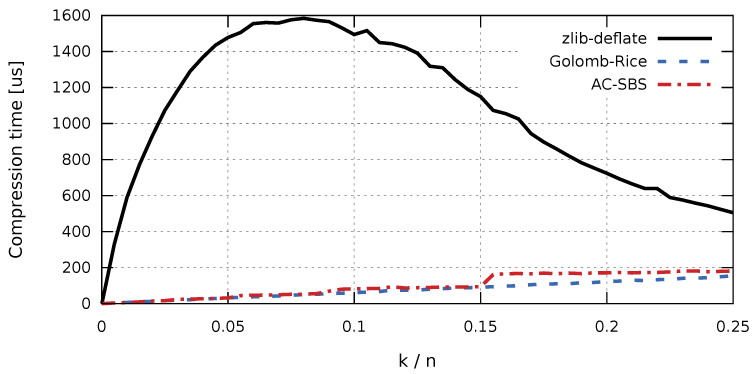
Compression time of test sequences (x86 architecture).

In the case of collecting production data for the purpose of future analyzes, aimed at, for example, improving the quality of products, access time to the stored data sequences is important. The factor that largely determines fast access to historical data is the time of sequence decompression. The access time affects not only the convenience of working with data, but also the cost of data processing (e.g., the cost of cloud computing). Due to the comparable sizes of compressed sequences, the difference in decompression times will be significant for the Golomb-Rice and AC-SBS algorithms. Sequence decompression times were examined independently for the ARM (RISC) architecture (Figure 6) and x86 (CISC) architecture (Figure 7). For completeness, graphs of compression times for the compared algorithms are also presented. Compression times as a function of k/n were also examined independently for the ARM (RISC) architecture (Figure 8) and for the x86 (CISC) architecture (Figure 9). However, the compression process itself will not be analyzed in detail. This is due to the fact that, for the interesting k/n intervals, the compression time for both Golomb-Rice and AC-SBS is similar.

**Figure 10:**
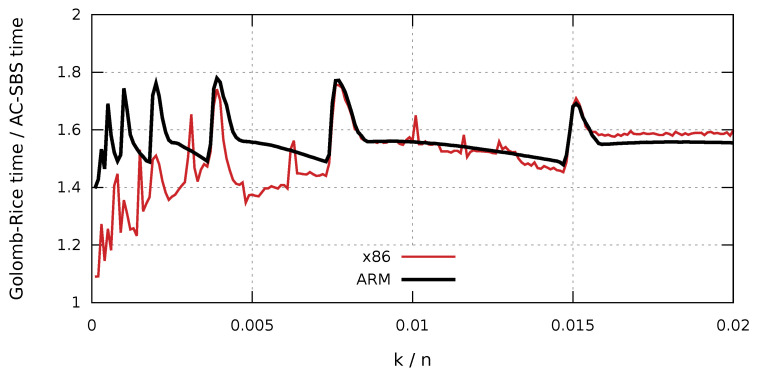
Ratio of sequence decompression times by Golomb-Rice and AC-SBS methods—ARM and x86 architecture.

The results presented in Figure 6 and Figure 7 clearly show that, in both hardware configurations, the zlib-deflate algorithm achieves by far the longest decompression time. For both types of processors, sequences created using the AC-SBS method are decompressed the fastest. For k/n changing from 0 to 0.02, Golomb-Rice decompression time exceeds AC-SBS decompression time by an average of 56.1% in ARM architecture and by 50.9% in x86 architecture. Figure 10 shows the ratio of sequence decompression times by the Golomb-Rice and AC-SBS methods for k/n changing from 0 to 0.02 for x86 and ARM architecture.

**Figure 11:**
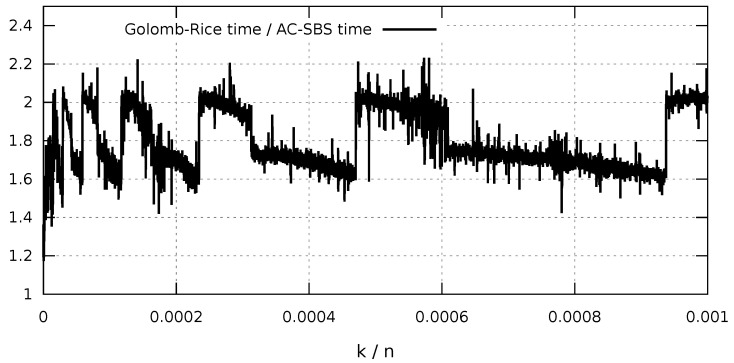
Ratio of decompression times by Golomb-Rice and AC-SBS methods in x86 architecture.

In addition, a comparison of data decompression speed was made for data sequences containing $n=10^{8}$ elements. Such research is justified by the possibility of using such extensive data sets, e.g., to analyze problems of product quality in mass production [3]. Figure 11 shows the ratio of decompression times using the Golomb-Rice method and the AC-SBS algorithm. It illustrates the results of an analysis of decompression of sparse data sets (k/n≤0.001) in x86 architecture. The presented results confirm that decompression efficiency using the AC-SBS algorithm is even higher. Golomb-Rice decompression lasts approximately 1.5 to 2 times longer compared with AC-SBS. The abrupt changes in the time ratio visible in the Figure 11 result from the automatic codeword length selection mechanism used in both compression methods.

**Figure 12:**
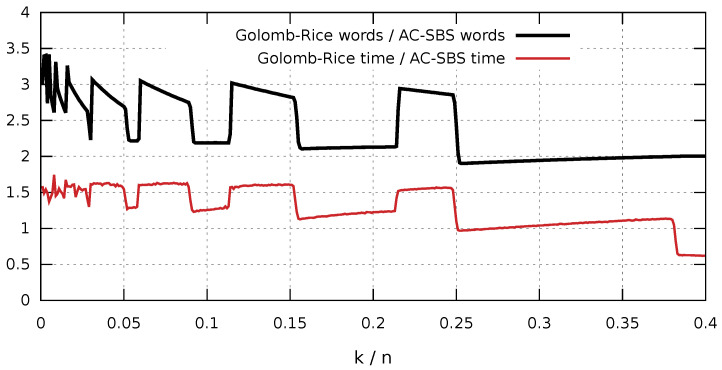
Correlation between the x86 decompression rate ratio and the ratio of the number of Golomb-Rice codewords and the number of AC-SBS codewords.

To end this section, we would like to discuss one more relationship mentioned at the end of the section dedicated to the AC-SBS algorithm, namely the correlation between the ratio of the number of codewords and the ratio of decompression rate. Figure 12 shows two graphs: the first one (solid line) shows the ratio of the number of Golomb-Rice codewords to the number of AC-SBS codewords, while the second one shows the ratio of Golomb-Rice coding decompression rate to AC-SBS decompression rate. At this point, we would like to point out that we assume a Golomb-Rice codeword to be any sequence of bits in which processing involves the execution of a conditional instruction. We explain this fact to avoid ambiguous interpretations. For example, we assume that a sequence of 10 zeros encoded using a binary sequence 11010 consists of four codewords 1|1|0|10. It clearly shows from the graphs that there is a certain relationship between the analyzed values. It *breaks down* when the k/n ratio is higher than 0.38, but this results from the fact that, in such a case, both methods fall into their degenerate form in which effective compression is basically impossible. The determined correlation coefficient for k/n<0.38 is 0.946 for the x86 and 0.957 for ARM architecture. Based on the obtained results, we can conclude that the number of conditional instructions in the decompression method directly affects its implementation time.

## 4. Discussion

In the article, we estimate the entropy of *n*-bit binary sequences with exactly *k* terms equal to one. We used the proven inequalities as the starting point for a new binary sequence compression algorithm to be applied mainly for coding long series and inverted indexes. The AC-SBS algorithm presented in the third part of the article is up to 8% less efficient than the Golomb-Rice algorithm in terms of compression. On the other hand, the speed of decompression attained with the use of the AC-SBS algorithm can by even twice higher compared with the Golomb-Rice method. The graphs presented in the article clearly show that the relationship between the two methods strongly depends on the value of the k/n ratio (the probability of ones appearing in the sequence). We can definitely say, however, that there are such k/n values for which AC-SBS is an attractive method of storing inverted indexes. The presented results clearly show that the speed of compression and decompression for both AC-SBS and Golomb-Rice depends stepwise on the optimal length of the codeword for a given sequence. The characteristic *steps* on the speed graphs appear each time when there is a change in the codeword length being the major determinant of the computational complexity of the entire process. It is also noteworthy that universal compression algorithms (such as the Lempel-Ziv algorithm) differ markedly in terms of both compression and speed from methods dedicated to coding long series and inverted indexes. The difference is so large that it practically rules out the use of universal methods in such applications. The algorithm presented in the article has a practical value. It has already been used by the authors in one of the commercial projects to reduce energy consumption. The project was carried out on a microcontroller with low computing power and assumed the replacement of Golomb-Rice decompression with AC-SBS in order to further reduce energy demand.

## Figures and Tables

**Table 1:** Relative sequence sizes for Golomb-Rice and AC-SBS compression methods compared to entropy.

k/n	0.0005	0.001	0.002	0.005	0.01	0.02	0.05
ZLIB/ENT	5.051	3.676	3.000	2.415	1.969	1.691	1.458
ACSBS/ENT	1.254	1.180	1.132	1.095	1.085	1.077	1.092
RICE/ENT	1.237	1.117	1.068	1.029	1.017	1.011	1.014
